# Incremental values of AOPP, IL-6, and GDF15 for identifying arteriosclerosis in patients with obstructive sleep apnea

**DOI:** 10.1186/s40001-024-01723-9

**Published:** 2024-02-20

**Authors:** Xinxin Li, Wen Liu, Yonghuai Wang, Cuiting Zhao, Qing Zhu, Zhishuang Dong, Chunyan Ma

**Affiliations:** 1 Department of Cardiovascular Ultrasound, The First Hospital of China Medical University, No. 155 Nanjingbei Street, Shenyang, 110001 Liaoning China; 2 Key Laboratory of Diagnostic Imaging and Interventional Radiology, Shenyang, Liaoning China; 3 Clinical Medical Research Center of Imaging in Liaoning Province, Shenyang, Liaoning China

**Keywords:** Obstructive sleep apnea, Arteriosclerosis, Oxidative stress, Inflammation, Growth differentiation factor 15

## Abstract

**Background:**

The objective of this study was to determine the independent and incremental values of advanced oxidative protein product (AOPP), interleukin 6 (IL-6), and growth differentiation factor 15 (GDF15) in identifying arteriosclerosis in patients with obstructive sleep apnea (OSA).

**Methods:**

A total of 104 individuals diagnosed with OSA by polysomnography were recruited in our study. Arteriosclerosis was defined by measuring the ultrafast pulse wave velocity of the carotid artery. Peripheral venous blood samples were collected to analyze the levels of AOPP, IL-6, and GDF15 utilizing commercially available enzyme-linked immunosorbent assays.

**Results:**

Compared to OSA patients without arteriosclerosis, those with arteriosclerosis exhibited significantly higher levels of AOPP, IL-6, and GDF15. GDF15 remained significantly associated with arteriosclerosis even after accounting for clinical factors such as age, gender, body mass index, systolic blood pressure, fasting blood glucose, smoking, and the apnea–hypoxia index (AHI). GDF15 demonstrated the largest area under the curve (AUC) for identifying arteriosclerosis in OSA patients (AUC, 0.85 [0.77–0.94]). The logistic regression model, combining clinical factors and AHI, was enhanced by the inclusion of AOPP and IL-6 (Chi-square = 25.06), and even further improved when GDF15 was added (Chi-square = 50.74). The integrated discrimination index increased by 0.06 to 0.16 when GDF15 was added to the models including clinical factors, AOPP, and IL-6.

**Conclusions:**

This study verified the independent and incremental value of GDF15 in identifying arteriosclerosis in OSA patients, surpassing clinical risk factors and other serum biomarkers such as AOPP and IL-6.

**Supplementary Information:**

The online version contains supplementary material available at 10.1186/s40001-024-01723-9.

## Background

Obstructive sleep apnea (OSA) is a prevalent sleep disorder characterized by recurring episodes of apnea and hypoventilation during sleep [1, 2]. OSA is closely linked to various cardiovascular diseases (CVD), including hypertension and coronary artery disease, and can even lead to severe cardiovascular events like cardiac arrest [3–5]. Studies have consistently shown the presence of arteriosclerosis in patients with OSA [6–8]. Furthermore, even after adjusting for confounding factors, including comorbidities, the severity of OSA remained significantly associated with arterial stiffness [9]. Arteriosclerosis is an important biomarker for assessing the risk of CVD in the general population [10]. Moreover, the presence of increased arterial stiffness can be an early indicator of future vascular complications in individuals with untreated moderate-to-severe OSA who do not have overt CVD [11]. Therefore, elucidating the pathophysiology of arteriosclerosis associated with OSA and identifying novel targets for diagnosis and treatment will contribute to reducing the morbidity and mortality of CVD in patients with OSA.

Numerous studies have demonstrated that OSA creates an independent environment conducive to the production of free radicals and inflammation [12]. Reactive oxygen species target plasma proteins, resulting in the formation of advanced oxidative protein product (AOPP), a hallmark of oxidative stress [13, 14]. Multiple studies have consistently shown significantly higher levels of AOPP in patients with OSA compared to healthy individuals [15, 16]. Increased oxidative stress can disrupt the bioavailability of nitric oxide in the bloodstream, further contributing to the development of arteriosclerosis [17]. However, the relationship between AOPP and arteriosclerosis in patients with OSA remains unexplored. Investigations have revealed heightened levels of the pro-inflammatory cytokine interleukin 6 (IL-6) in patients with OSA [18, 19]. Evidence also suggests that IL-6 contributes to the development of athero-/arteriosclerosis through its chronic low-grade inflammatory effects [20]. Consequently, we hypothesized that IL-6 may play a role in the identification of arteriosclerosis in patients with OSA.

Growth differentiation factor 15 (GDF15) belongs to the transforming growth factor beta superfamily and plays a crucial role in vascular development and remodeling [21]. In response to external stimuli, such as hypoxic, oxidative, or inflammatory stress, GDF15 exhibits a robust upregulation [22]. Previous research has linked GDF15 to atherosclerotic CVD events and overall mortality [23]. A study by Kamran Sari et al*.* did not observe elevated GDF15 levels in OSA patients compared to controls. This may be due to the fact that the proportion of patients with severe OSA was only 40% in the OSA group [24]. Therefore, there is indeed a need to further explore the relationship between OSA and GDF15. Notably, GDF15 was found to be associated with age [24–26]. Arterial stiffness increases with age, and we therefore hypothesized that GDF15 could serve as a biomarker of arteriosclerosis in patients with OSA.

Herein, the current study tried 1) to assess the predictive value of AOPP, IL-6, and GDF15 in relation to arteriosclerosis in patients with OSA; and 2) to examine the incremental value of AOPP, IL-6, and GDF15 in identifying arteriosclerosis in patients with OSA.

## Materials and methods

### Study population

This case–control study was carried out at the Department of Respiratory Medicine of the First Hospital of China Medical University. Between March 2021 and March 2022, a consecutive group of participants who exhibited symptoms like snoring and daytime sleepiness, were diagnosed with OSA by polysomnography, and volunteered to take part. The OSA diagnosis was based on the criteria of the American Academy of Sleep Medicine (2012) [27]. Various exclusion criteria were applied, including under 18 years of age, central sleep apnea, a history of stroke, coronary artery disease, previous OSA treatment, hepatic or renal impairment, malignancy, autoimmune or inflammatory diseases, acute or chronic vascular inflammation, abnormal thyroid function, use of antidepressants or sedative drugs, and carotid atherosclerotic plaque formation. Informed consent was obtained from all patients prior to hospital admission. The study was approved by the China Medical University Ethics Committee and adhered to the principles outlined in the Declaration of Helsinki.

### OSA diagnosis by polysomnography

Participants underwent polysomnography using the Embla system (Natus, Pleasanton, CA) to record various nocturnal sleep characteristics, including blood oxygen saturation (SpO_2_), airflow, posture, snoring sound, and chest and abdomen movement during breathing. The apnea–hypopnea index (AHI) was calculated as the number of apneas and hypoventilations per hour of sleep, with mild, moderate, and severe OSA defined as 5 ≤ AHI < 15, 15 ≤ AHI < 30, and AHI ≥ 30, respectively. Sleep apnea was defined as a 90% decrease in oral and nasal airflow from baseline lasting for a minimum of 10 s, while hypoventilation was defined as a 30–90% reduction in oral and nasal airflow lasting for at least 10 s, accompanied by a decrease in SpO_2_ of at least 4%. The clinician responsible for conducting the polysomnography was blinded to the participants' group allocation and any other test results.

### Carotid ultrasound imaging

**Fig. 1:**
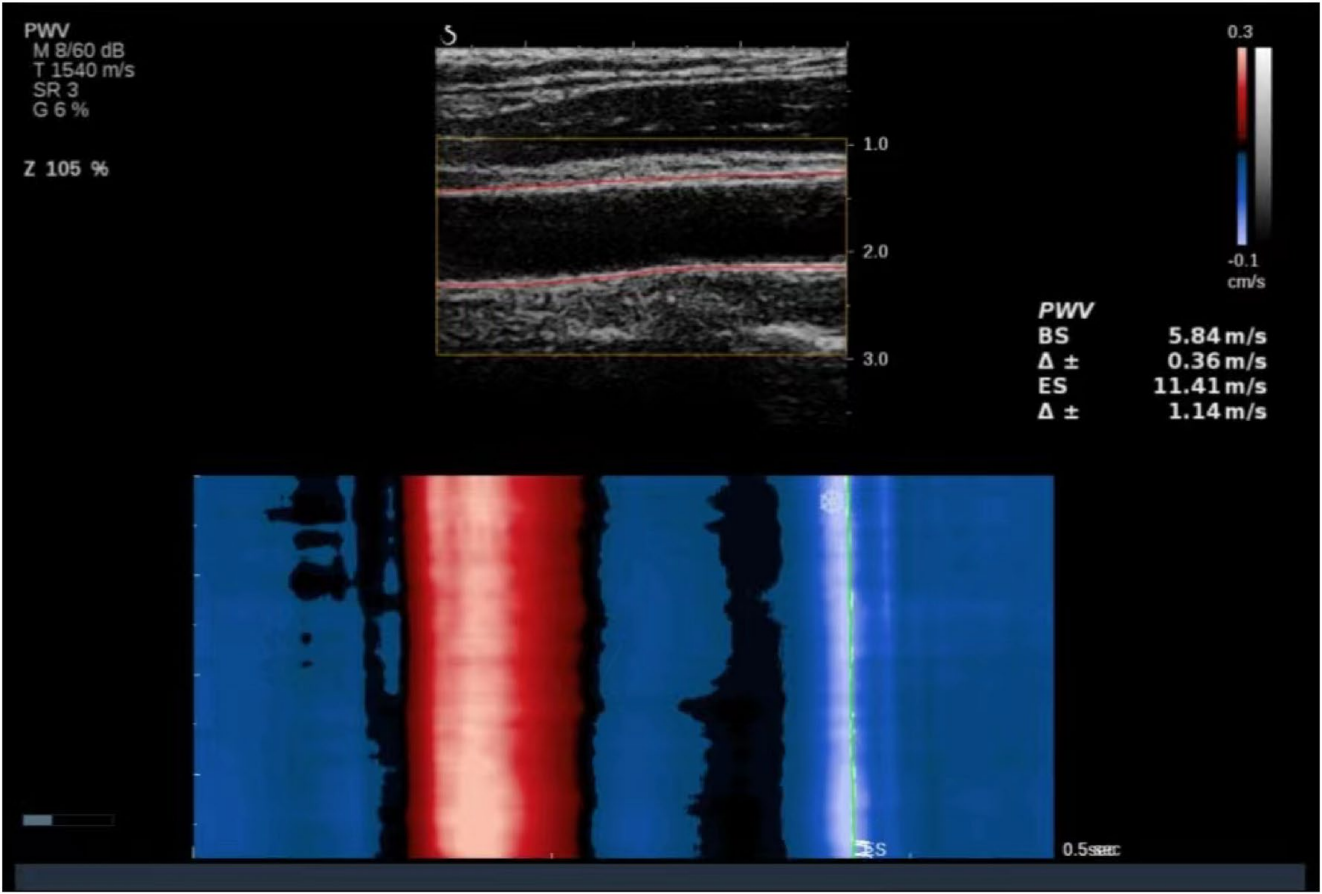
Representative images of PWV measurements at ES and BS using ultrafast ultrasound imaging. The yellow rectangular box represents the area of interest. The two red lines represent the anterior and posterior walls of the auto-tracked common carotid artery. The lumen between the two red lines is the carotid artery. The software can automatically identify and record PWV at BS and ES. The Δ ± values indicated the variance in PWV measurements and should be kept below 20% of the PWV values

24 h after diagnosis of OSA, participants underwent standardized carotid ultrasonography examinations using an Aixplorer ultrasound system equipped with the SL 10–2 probe (SuperSonic Imagine, France). The measurements were performed following the guidelines for peripheral arterial disease set by the European Society of Cardiology [28]. The longitudinal images of the common carotid artery (CCA) were collected at 1 cm proximal to the carotid bifurcation. The probe was adjusted to ensure clear visibility of both the anterior and posterior walls of the CCA. Carotid intima–media thickness (cIMT) was measured, and shear wave elastography was then obtained at the same location as the longitudinal CCA image. For the superficial walls of the CCA, the pulse wave velocity_beginning of systole (PWV_BS) and pulse wave velocity_end of systole (PWV_ES) were collected. As shown in Fig. 1, the software automatically recognized and recorded the PWV at both the BS and ES. The Δ ± values represented the variability in PWV measurements, which needed to be maintained below 20% of the PWV values. Furthermore, three consecutive measurements were taken at the same site, and a mean value was calculated. Arteriosclerosis is defined as an increase in arterial stiffness, measured by ufPWV, with reference values stratified by age and gender based on our previous research [29].

### Blood sample collection and postprocessing

After carotid ultrasound measurements, peripheral venous blood samples were collected from participants within 12 h. The samples were then subjected to centrifugation at 3000 rpm for 10 min to separate the serum, which was subsequently stored at −80 °C until further analysis. The levels of alanine transaminase (ALT), creatinine, urea, triglycerides, total cholesterol, low-density lipoprotein cholesterol (LDLC), high-density lipoprotein cholesterol (HDLC), and fasting blood glucose (FBG) were measured using a Siemens ADVIA 2400 analyzer (NY, USA). Additionally, the levels of advanced oxidation protein products (AOPP), IL-6, and GDF15 were determined using commercial Enzyme‐linked Immunosorbent Assay Kits (MEIMIAN Biological Technology, China).

### Reproducibility

Ten OSA patients were randomly selected to evaluate the intra- and inter-observer variability of PWV_ES and PWV_BS. For intra-observer variability assessment, the same observer, unaware of the initial measurements, performed the same measurements again over the course of four weeks. To evaluate inter-observer variability, two observers independently repeated the measurements twice.

### Statistical analysis

Continuous data were reported as mean ± standard deviation or median (interquartile range), while categorical data were presented as counts or percentages. The normality of the data was assessed using the Shapiro–Wilk test. Student's *t*-test was utilized for normally distributed continuous variables, while the Mann–Whitney *U*-test was employed for variables that did not meet the assumption of normality. The Chi-square test was applied to compare categorical variables. Correlation analysis between the two variables used Pearson correlation analysis. Logistic regression analysis was performed to identify independent predictors of arteriosclerosis in individuals with OSA. The results were reported as odds ratios (OR) with corresponding 95% confidence intervals (CI). Receiver operating characteristic (ROC) analysis was conducted to evaluate the predictive value of selected variables and their combinations for arteriosclerosis in OSA patients, with the area under the curve (AUC) calculated to assess discriminatory power.

The incremental benefits of the models were assessed by calculating the improvement in the Chi-square statistic and the Cox and Snell *R*^2^, or Nagelkerke *R*^2^. To evaluate the additional value of serum biomarkers to the original model, the categorical net reclassification improvement (^c^NRI) and integrated discrimination index (IDI) were computed. Intra- and inter-observer reproducibility, as well as bias and limits of agreement (LOA) between measurements, were estimated using Bland–Altman analysis. Data analysis and graph generation were performed using SPSS version 25 (IBM SPSS Statistics for Windows, Version 25.0, USA) and R version 4.2.1 (R Foundation for Statistical Computing, Vienna, Austria). *P* < 0.05 was deemed statistically significant.

## Results

### Clinical characteristics and laboratory examinations

A total of 104 OSA patients were included in the study, 28 (27%) of whom were diagnosed with arteriosclerosis as evidenced by increased arterial stiffness. Based on the presence or absence of arteriosclerosis, the OSA patients were categorized into two groups: OSA with arteriosclerosis and OSA without arteriosclerosis. Table 1 presents the comparison of clinical characteristics and laboratory examinations between the two groups. The results revealed no significant differences between the groups in terms of age, gender, height, weight, and body mass index. However, it was observed that systolic blood pressure (SBP, 129 vs. 124 mmHg, *p* = 0.02) and diastolic blood pressure (DBP, 88 vs. 78, *p* = 0.04) were significantly higher in the OSA patients with arteriosclerosis. Regarding laboratory parameters, no significant differences were found between the two groups in FBG, ALT, creatinine, urea, triglycerides, total cholesterol, HDLC, and LDLC. Conversely, elevated levels of AOPP (58 vs. 56 μmol/L, *p* = 0.01), IL-6 (59 vs. 58 pg/mL, *p* = 0.03), and GDF15 (101 vs. 94 ng/mL, *p* = 0.01) were observed in the OSA patients with arteriosclerosis compared to those without arteriosclerosis.

**Table 1:** Comparison of basic characteristics of patients with OSA according to the presence of arteriosclerosis

	All patient (*n* = 104)	OSA without arteriosclerosis (* n* = 76)	OSA with arteriosclerosis (* n* = 28)	*p* value
Clinical characteristics				
Age (yrs)	44.06 ± 10.37	44.37 ± 10.02	43.21 ± 11.41	0.62
Male (n/%)	78 (75%)	58 (76%)	20 (71%)	0.61
Height (cm)	172.12 ± 8.32	172.17 ± 7.94	171.96 ± 9.43	0.91
Weight (kg)	83.94 ± 14.40	84.01 ± 14.19	83.61 ± 15.24	0.92
Body mass index (kg/m2)	28.31 ± 4.33	28.33 ± 4.38	28.27 ± 4.28	0.95
Systolic blood pressure (mmHg)	130.96 ± 14.31	124.25 ± 7.18	129.05 ± 13.93	**0.02**
Diastolic blood pressure (mmHg)	86.54 ± 8.90	78.20 ± 6.04	87.82 ± 8.46	**0.04**
Smoking (n/%)	57 (55%)	39 (51%)	18 (64%)	0.24
Laboratory examination				
Fasting blood glucose (mmol/L)	5.25 ± 0.60	5.27 ± 0.64	5.20 ± 0.45	0.61
ALT (U/L)	21.00 (18.00–25.00)	21.50 (18.00–26.00)	21.00 (18.00–25.00)	0.91
Creatine (μmol/L)	69.73 ± 11.59	68.65 ± 11.74	72.64 ± 10.84	0.12
Urea (mmol/L)	4.87 ± 1.20	4.87 ± 1.22	4.87 ± 1.16	0.98
Triglyceride (mmol/L)	1.15 (1.01–1.38)	1.13 (1.01–1.38)	1.23 (0.83–1.58)	0.70
Total cholesterol (mmol/L)	4.43 ± 1.02	4.39 ± 0.96	4.52 ± 1.19	0.57
HDLC (mmol/L)	1.24 ± 0.35	1.22 ± 0.34	1.26 ± 0.36	0.61
LDLC (mmol/L)	2.45 ± 0.89	2.41 ± 0.77	2.54 ± 1.16	0.49
AOPP (μmol/L)	56.60 (54.05–58.36)	56.35 (53.64–58.14)	57.89 (55.82–58.82)	**0.01**
IL-6 (pg/mL)	58.58 (56.10–59.97)	58.32 (55.59–59.98)	59.44 (57.13–61.30)	**0.03**
GDF15 (ng/L)	97.33 (93.35–100.76)	94.20 (92.21–98.66)	101.45 (99.33–103.68)	**0.01**

Data are presented as mean ± SD, median (interquartile range), or frequency (percentages). Bold value means *p* < 0.05

*ALT* alanine transaminase, *HDLC* high-density lipoprotein cholesterol, *LDLC* low-density lipoprotein cholesterol, *AOPP* advanced oxidation protein products, *IL-6* interleukin-6, *GDF15* growth differentiation factor 15

### Carotid ultrasound and sleep characteristics

Table 2 provides a comparison of carotid ultrasound findings and sleep characteristics between OSA patients with and without arteriosclerosis. Of 104 patients with OSA, 79 patients with AHI ≥ 30 were severe, 25 patients with AHI < 30 were mild–moderate. Arteriosclerosis was observed in 23 out of 79 (29%) patients with severe OSA, while it was observed in 5 out of 25 (20%) patients with mild-to-moderate OSA. Although the ratio of arteriosclerosis in severe OSA patients was higher than that in mild–moderate OSA patients, there was no significant difference between two groups (29% vs. 20%; *p* = 0.45). The OSA patients with arteriosclerosis exhibited higher values of PWV_ES (9.43 vs. 6.89; *p* = 0.01) and PWV_BS (6.79 vs. 5.34; *p* = 0.01). Furthermore, the OSA patients with arteriosclerosis had significantly higher AHI values (64.51 vs. 51.08; *p* = 0.02) compared to those without arteriosclerosis. However, there were no significant differences between the two groups in terms of mean apnea duration, longest apnea duration, lowest SpO_2_, and mean SpO_2_.

**Table 2:** Comparison of serum biomarkers of patients with OSA according to the presence of arteriosclerosis

	All patient (* n* = 104)	OSA without arteriosclerosis (* n* = 76)	OSA with arteriosclerosis (* n* = 28)	*p* value
Carotid ultrasound				
PWV_ES (m/s)	5.73 ± 1.25	6.89 ± 1.41	9.43 ± 2.04	**0.01**
PWV_BS (m/s)	7.57 ± 1.95	5.34 ± 0.91	6.79 ± 1.42	**0.01**
Carotid IMT (mm)	0.56 (0.50–0.64)	0.56 (0.52–0.62)	0.57 (0.49–0.68)	0.84
Sleep characteristics				
AHI (events/h)	54.69 ± 27.12	51.08 ± 26.36	64.51 ± 21.19	**0.02**
Mean apnea duration (s)	23.57 ± 8.90	26.32 ± 7.83	28.29 ± 10.96	0.55
Longest apnea duration (s)	50.40 ± 26.35	66.59 ± 25.30	61.71 ± 17.63	0.18
Lowest SpO_2_ (%)	75.65 ± 12.06	70.05 ± 13.80	73.83 ± 14.40	0.43
Mean SpO_2_ (%)	92.48 ± 4.54	89.05 ± 6.63	91.07 ± 4.12	0.66
SIT90 (%)	13.01 ± 17.32	11.97 ± 13.87	13.55 ± 19.00	0.75
OSA Severity				0.45
Mild–moderate (n/%)	25 (24%)	20 (80%)	5 (20%)	
Severe (n/%)	79 (76%)	56 (71%)	23 (29%)	

Data are presented as mean ± SD or median (interquartile range). Bold value means *p* < 0.05

*PWV_BS* pulse wave velocity at the beginning of systole, *PWV_ES* pulse wave velocity at the end of systole, *IMT* intima–media thickness, *AHI*, apnea–hypopnea index, *SpO*_*2*_ blood oxygen saturation, *SIT90* ratio of time with SpO_2_ below 90% in total sleep time

Correlations of the serum biomarkers (including AOPP, IL-6 and GDF15) with AHI and ufPWV were performed by Pearson correlation analyses (Additional file 1: Figure S1). The results showed that AOPP, IL-6, and GDF15 are all positively correlated with AHI and PWV_ES, which illustrated that the serum biomarkers are not only related to OSA but also to arteriosclerosis.

### Reproducibility

**Fig. 2:**
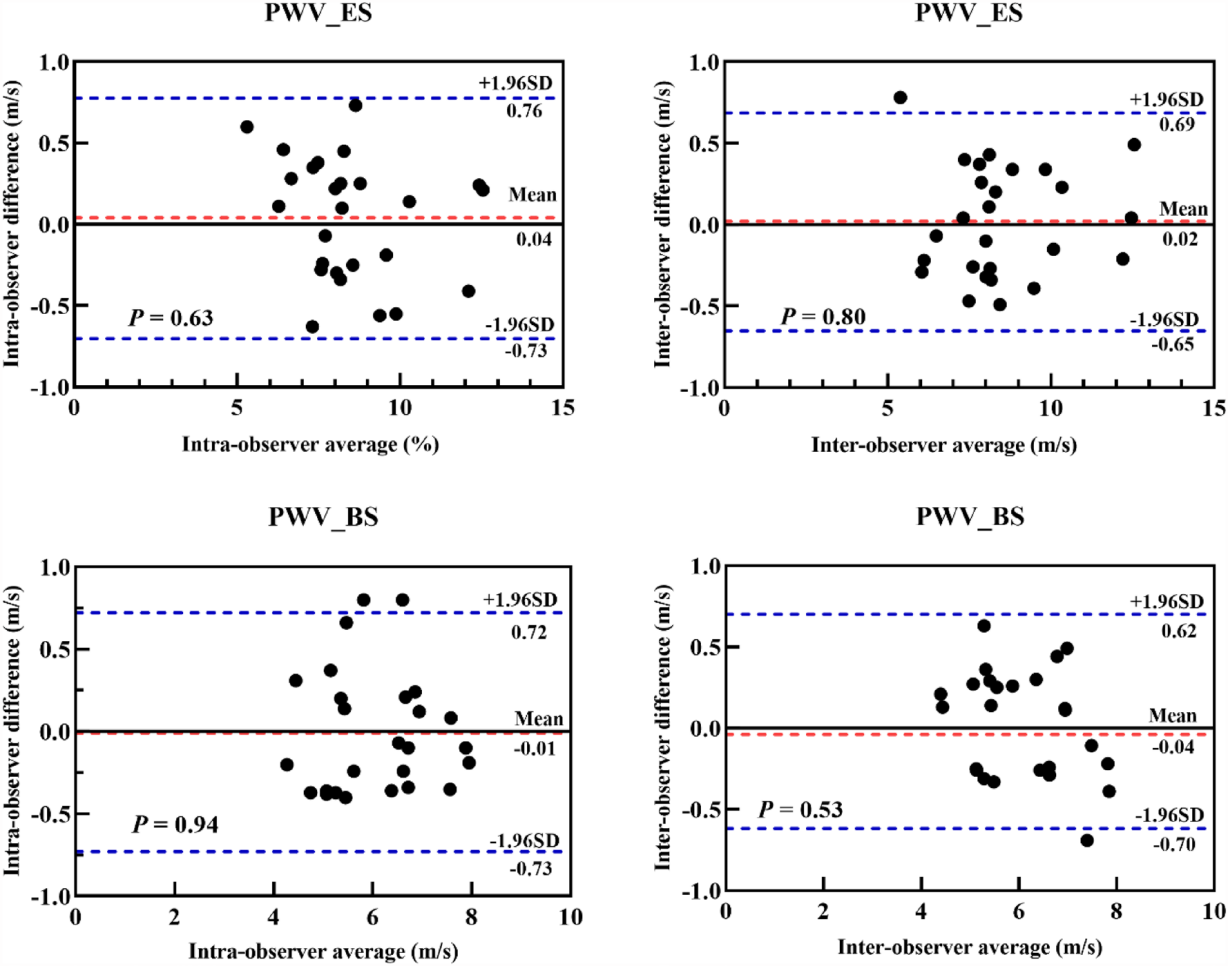
Bland–Altman analysis for the intra-observer and inter-observer variabilities of PWV_ES and PWV_BS. Abbreviations: PWV_BS, pulse wave velocity at the beginning of systole; PWV_ES, pulse wave velocity at the end of systole

The Bland–Altman plots demonstrated excellent intra- and inter-observer reproducibility for the PWV_ES measurement, with no significant biases observed for both intra-observer (0.04 m/s [−0.76–0.73 m/s]; *p* = 0.63) and inter-observer (−0.02 m/s [−0.65–0.69 m/s]; *p* = 0.80) analyses. Similarly, no significant biases were found for the PWV_BS measurement in intra-observer (−0.01 m/s [−0.73–0.72 m/s]; *p* = 0.94) or inter-observer (−0.04 m/s [−0.70–0.62 m/s]; *p* = 0.53) analyses (Fig. 2). These results indicated strong consistency in the measurement of PWV_ES and PWV_BS between observers and within multiple measurements by the same observer.

### Predictors of arteriosclerosis

**Fig. 3:**
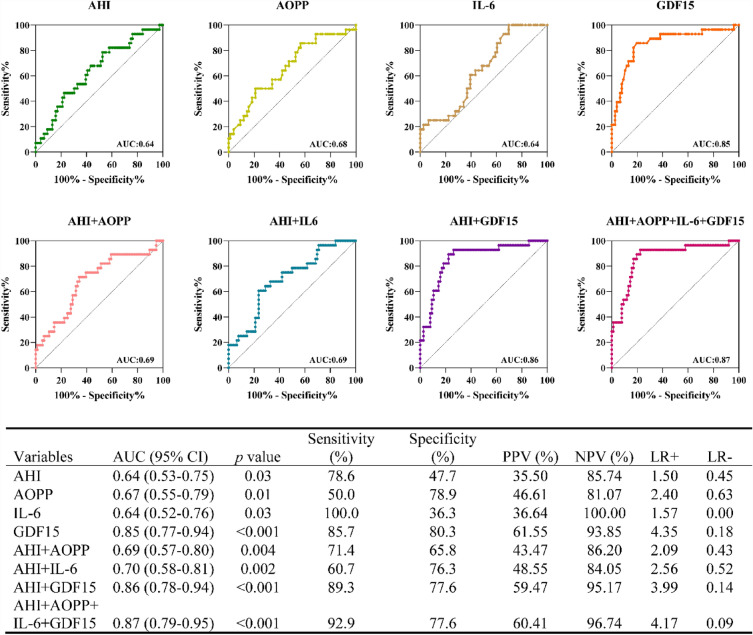
Receiver operating characteristic analysis of serum biomarkers to identify arteriosclerosis in patients with OSA. AHI, apnea–hypopnea index; AOPP, advanced oxidation protein products; IL-6, interleukin-6; GDF15, growth differentiation factor 15; AUC, area under curve; CI, confidence interval; PPV, positive predictive value; NPV, negative predictive value; LR ( +), positive likelihood ratio; LR (−), negative likelihood ratio

In the univariable logistic analysis presented in Table 3, arterial stiffness showed significant associations with several factors, including SBP (OR [95%CI] = 1.04 [1.00–1.07]; *p* = 0.03), DBP (OR [95%CI] = 1.05 [1.00–1.11]; *p* = 0.03), AHI (OR [95%CI] = 1.02 [1.00–1.04]; *p* = 0.03), AOPP (OR [95%CI] = 1.26 [1.05–1.52]; *p* = 0.01), IL-6 (OR [95%CI] = 1.21 [1.05–1.40]; *p* = 0.01) and GDF15 (OR [95%CI] = 1.45 [1.23–1.70]; *p* < 0.01). An analysis of the ROC curve for predicting arteriosclerosis is presented in Fig. 3. The results revealed that AHI, AOPP, IL-6, and GDF15 were significant predictors of arteriosclerosis. Notably, GDF15, specifically in patients with OSA, exhibited a higher AUC of 0.85 [0.77–0.94] compared to AOPP (AUC = 0.67 [0.55–0.79]) or IL-6 (AUC = 0.64 [0.52–0.76]) in detecting arteriosclerosis, suggesting that GDF15 had a superior performance as a serum biomarker for identifying arteriosclerosis compared to the other mentioned biomarkers. Additionally, in patients with OSA, the combination of AHI, AOPP, IL-6, and GDF15 yielded the highest AUC (0.87 [0.79–0.95]), demonstrating a sensitivity of 92.9% and specificity of 77.6% in identifying arteriosclerosis.

**Table 3:** Univariable logistics regression analysis for identifying the presence of arteriosclerosis in patients with OSA

	OR [95%CI]	*p* value
Age (yrs)	0.99 [0.95–1.03]	0.61
Gender	0.83 [0.32–2.11]	0.69
Body mass index (kg/m2)	1.00 [0.90–1.10]	0.95
Systolic blood pressure (mmHg)	1.04 [1.00–1.07]	**0.03**
Diastolic blood pressure (mmHg)	1.05 [1.00–1.11]	**0.04**
Fasting blood glucose (mmol/L)	0.82 [0.39–1.73]	0.61
Smoking	1.71 [0.70–4.18]	0.24
Apnea–hypopnea index (events/h)	1.02 [1.00–1.04]	**0.03**
AOPP (μmol/L)	1.26 [1.05–1.52]	**0.01**
IL-6 (pg/mL)	1.21 [1.05–1.40]	**0.01**
GDF15 (ng/L)	1.45 [1.23–1.70]	**0.01**

Bold value means *p* < 0.05

*AOPP* Advanced oxidation protein products, *IL-6* interleukin-6, *GDF15* growth differentiation factor 15, *OR* odds ratio, *CI* confidence interval

### Arteriosclerosis prediction models

**Fig. 4:**
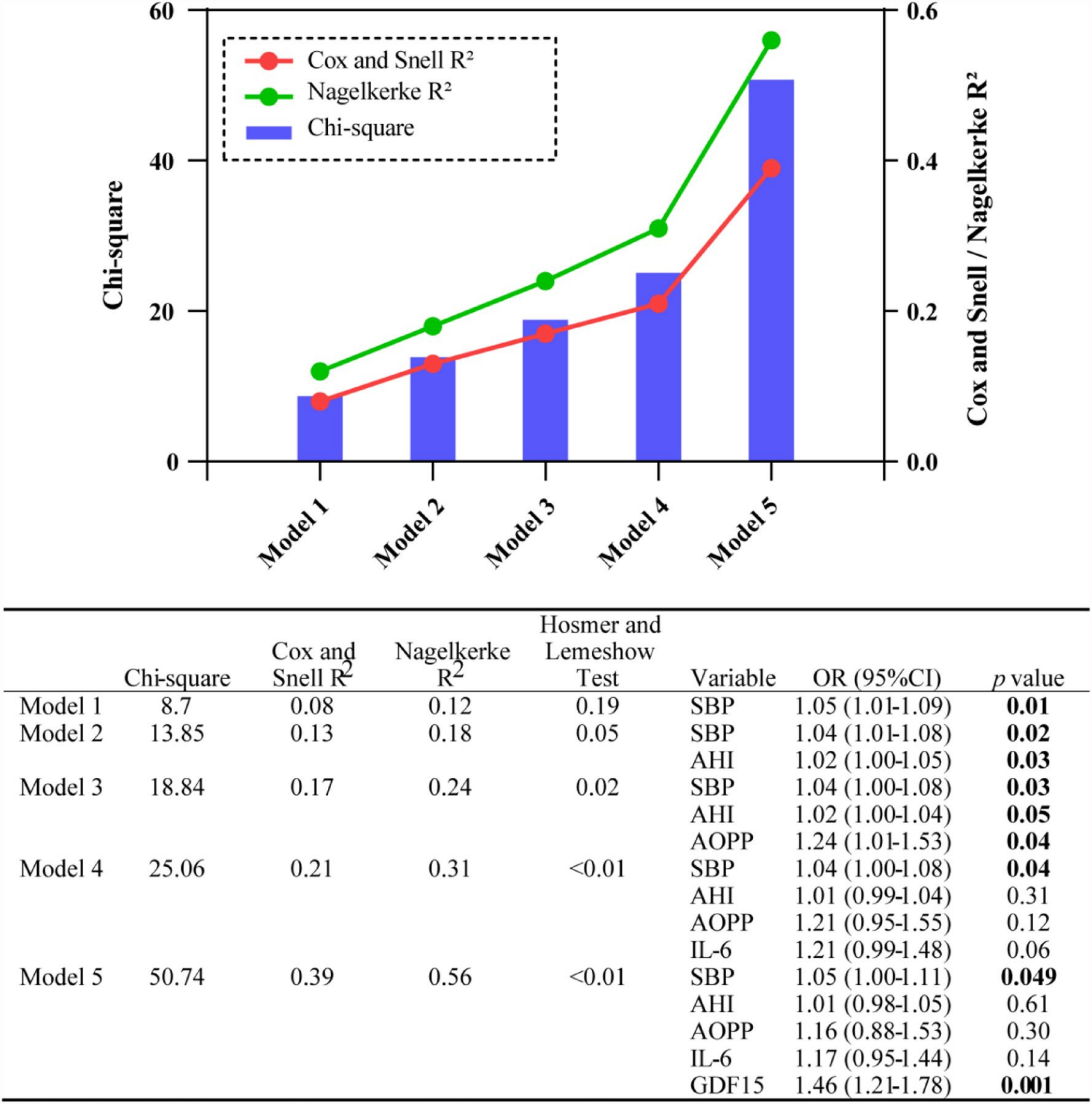
Incremental value of serum biomarkers for identifying arteriosclerosis in patients with OSA in the logistic regression models. Model 1 included age, gender, body mass index, SBP, FBG and smoking**;** Model 2 included Model 1 plus AHI; Model 3 included Model 2 plus AOPP; Model 4 included Model 3 plus IL-6; Model 5 included Model 4 plus GDF15. OR, odds ratio; CI, confidence interval; SBP, systolic blood pressure; FBG, fasting blood glucose; AHI, apnea–hypopnea index; AOPP, advanced oxidation protein products; IL-6, interleukin-6; GDF15, growth differentiation factor 15

Based on previous literature, clinical knowledge, and statistically significant covariates from the univariate analysis, a series of multivariable logistic models were examined to determine independent predictors of arteriosclerosis, as shown in Fig. 4. Model 1 is a clinical traditional risk factor-only model for predicting arteriosclerosis, including age, gender, body mass index, SBP, FBG and smoking [30]. Notably, a collinearity diagnostic showed that there was a collinear between SBP and DBP (Eigenvalue tends to be zero and Condition index > 10 in all dimensions, Additional file 1: Table S1), Therefore, only SBP was used in Model 1 in order to reduce the bias caused by the collinearity between SBP and DBP. SBP (OR [95%CI] = 1.05 (1.01–1.09); *p* < 0.01) emerged as an independent predictor in Model 1.

Next, backward stepwise logistic regression analysis was used to determine the sequence of markers adding to the model after clinical model (model 1). The results of backward stepwise analysis showed that the AHI and AOPP were sequentially excluded out of the model in the step 2 and 3 (Additional file 1: Table S2). Therefore, we used sequential nested models by adding AHI, AOPP, IL-6 and GDF15 sequentially for predicting arteriosclerosis in OSA patients. AHI remained an independent predictor in Model 2, which accounted for the variables in Model 1 and included AHI (model 2; OR [95%CI] = 1.02 (1.00–1.05); *p* < 0.03). AOPP, IL-6, and GDF15 were sequentially incorporated into Models 3–5. Among these, GDF15 exhibited an independent association with arteriosclerosis even after adjusting for age, gender, body mass index, SBP, FBG, smoking, AHI, AOPP, and IL-6 in Model 5 (OR [95%CI] = 1.46 (1.21–1.78); *p* < 0.03).

### Incremental value of serum biomarkers

As depicted in Fig. 3, the inclusion of AOPP increased the AUC of AHI for identifying arteriosclerosis from 0.64 (95% CI [0.53–0.75]) to 0.69 (95% CI [0.57–0.80]). Moreover, the addition of IL-6 further increased the AUC of AHI from 0.64 (95% CI [0.53–0.75]) to 0.70 (95% CI [0.58–0.81]). Notably, the combination of AHI and GDF15 demonstrated the highest incremental AUC of 0.86 (95% CI [0.78–0.94]) for accurately identifying arteriosclerosis. These findings indicated that incorporating serum biomarkers such as AOPP, IL-6, and GDF15 with AHI significantly enhanced the diagnostic value of arteriosclerosis in patients with OSA. Additionally, GDF15 exhibited greater incremental value compared to AOPP or IL-6 in the detection of arteriosclerosis in patients with OSA.

The model for predicting arteriosclerosis in patients with OSA (model 2) was initially based on clinical parameters such as age, gender, body mass index, SBP, FBG, smoking, and AHI. The model yielded a Chi-square value of 13.85, with Cox and Snell *R*^2^ = 0.13 and Nagelkerke *R*^2^ = 0.18. Subsequent improvements were made by including AOPP in model 3, resulting in a Chi-square value of 18.84, with Cox and Snell *R*^2^ = 0.17 and Nagelkerke *R*^2^ = 0.24. IL-6 was added to model 4, leading to a Chi-square value of 25.06, with Cox and Snell *R*^2^ = 0.21 and Nagelkerke *R*^2^ = 0.31. Furthermore, model 5 demonstrated further improvement by incorporating GDF15, resulting in a Chi-square value of 50.74, with Cox and Snell *R*^2^ = 0.39 and Nagelkerke *R*^2^ = 0.56 (Fig. 4). In addition, model 5 showed a higher AUC value of 0.90 than the other models, indicating it was the most powerful at identifying arteriosclerosis in patients with OSA (Additional file 1: Figure S2).

Additional file 1: Table S3 presents the incorporation of serum biomarkers (AOPP, IL-6, and GDF15) with clinical parameters (age, gender, body mass index, SBP, FBG, and smoking) to improve the reclassification of arteriosclerosis. Adding AOPP to model 2, which is based on AHI and clinical parameters, did not result in a significant improvement in reclassification. Similarly, adding IL-6 to model 3 did not show a significant improvement either. However, the inclusion of GDF15 in model 4, which combines clinical parameters, AHI, AOPP, and IL-6, led to a notable enhancement in reclassification. Specifically, the NRI increased from 0.04 (-0.11 to 0.18) to 0.31 (0.11 to 0.51), and the IDI increased from 0.06 (-0.01 to 0.12) to 0.16 (0.10 to 0.24).

## Discussion

The study yielded several significant findings. Firstly, AOPP, IL-6, and GDF15 levels were observed to be elevated in patients with OSA and arteriosclerosis in comparison to those with OSA alone. Secondly, GDF15 emerged as an independent predictor of arteriosclerosis in patients with OSA. Thirdly, GDF15 exhibited incremental value when compared to AOPP and IL-6 in the identification of arteriosclerosis among patients with OSA. This study is notably the first to investigate the incremental value of AOPP, IL-6, and GDF15 in detecting arteriosclerosis within the OSA population. These findings are of great importance as they provide potential new targets for diagnosing and treating arteriosclerosis associated with OSA, potentially leading to notable reductions in cardiovascular morbidity and mortality rates for patients with OSA.

Arteriosclerosis has been shown to be independently associated with cardiovascular events [31]. Detecting arteriosclerosis at an early stage, before the emergence of morphological changes such as intima–media thickening or plaque formation, could potentially prevent severe cardiovascular events. The measurement of pulse wave velocity (PWV) is recommended in the 2018 European guidelines as a means to assess arterial stiffness [32]. A novel and unique method for PWV measurement called ultrafast ultrasound imaging (ufPWV) has recently been developed, offering an exceptionally high image sampling rate of over 10,000 frames per second [33]. This technique enables real-time tracking and visualization of local pulse wave propagation, displaying good reproducibility [34]. Traditional PWV measurements, such as carotid-femoral PWV, determine the transit time of the pulse wave between carotid and femoral arteries, whereas ufPWV directly estimates local arterial wall stiffness [35]. In a previous study, we established reference values for carotid ufPWV in a Chinese population stratified by age and gender, which can be used to diagnose arteriosclerosis [29]. Consequently, carotid ufPWV was evaluated in individuals diagnosed with OSA to determine the presence of arteriosclerosis using the established carotid ufPWV reference values specific to their age and gender. Subsequently, participants were divided into two groups: OSA with arteriosclerosis and OSA without arteriosclerosis. In this study, the prevalence of atherosclerosis in the OSA population was approximately 27%. To our knowledge, it is the first study to use carotid ufPWV to assess the prevalence of arteriosclerosis in patients with OSA. A larger sample size is needed in future studies to validate arteriosclerosis prevalence in OSA patients.

OSA is characterized by increased sympathetic activity, oxidative stress, upregulation of redox-sensitive genes, and an inflammatory cascade. The primary treatment for patients with moderate-to-severe OSA is continuous positive airway pressure (CPAP). However, a study conducted by McEvoy et al. found no evidence supporting the idea that CPAP effectively prevents cardiovascular events in patients with moderate-to-severe OSA and pre-existing CVD [36]. This lack of effectiveness may be due to poor adherence to CPAP therapy among patients with OSA, as well as the limited impact of CPAP on metabolic processes [37]. The pathogenesis underlying OSA-associated CVD shows significant variability.

Previous researches had shown the connections between IL-6 and arteriosclerosis in an animal study [38], AOPP and arteriosclerosis in healthy individuals [39], and GDF15 and arteriosclerosis in the general population [40]. In this investigation, the levels of serum biomarkers AOPP, IL-6, and GDF15 were found to be higher in individuals with OSA and arteriosclerosis compared to those with OSA only. Furthermore, AOPP, IL-6, and GDF15 are all positively correlated with AHI and PWV_ES. This further illustrates the serum biomarkers are not only related to OSA, but also to arteriosclerosis.

Clinical models that incorporate risk factor data have been proposed as a method to classify cardiovascular risk. Our study revealed that serum biomarkers provided additional value in identifying arteriosclerosis in patients with OSA, surpassing the clinical models that consider age, gender, body mass index, SBP, FBG, smoking, and AHI. Specifically, GDF15, a serum biomarker, significantly improved the diagnostic accuracy of Model 4 for arteriosclerosis in patients with OSA (Chi-square increased from 25 to 50).

Previous research has proposed that GDF15 plays a role in regulating vascular proliferation, differentiation, remodeling, and inflammatory damage repair, thus establishing a strong association between GDF15 and the diagnosis and prognosis of various cardiovascular conditions [41, 42]. The results of our study also confirmed the predictive significance of GDF15 and provided evidence that GDF15 enhanced the ability to differentiate and reclassify patients with OSA more accurately, determining the presence or absence of arteriosclerosis. This observation stems from the finding that elevated levels of circulating GDF15 are linked to increased plaque accumulation and higher artery calcium scores [43, 44]. Furthermore, a study by Bonaterra et al. on GDF15 knockout mice demonstrated that GDF15 deficiency protected against atherosclerosis, suggesting that targeted suppression of GDF15 could hinder arterial stiffness progression [45]. Consequently, GDF15 may improve the estimation of the pretest probability of arteriosclerosis and present new targets for diagnosing and treating OSA-associated arteriosclerosis, further reducing CVD morbidity and mortality in individuals with OSA.

## Limitation

This study had several limitations. Firstly, the small sample size of the study population can be attributed to the stringent exclusion criteria used to select participants. Additionally, the study was limited to a single-center cohort, necessitating a prospective multicenter trial to enhance the validity of the findings. Another limitation was the measurement of blood pressure within 5 min before the carotid scan, rather than simultaneously, which may impact the assessment of arterial stiffness. Furthermore, the definition of arteriosclerosis was based on Chinese patients, which restricts the generalizability of the results to other races or ethnicities. Therefore, further validation of these findings in diverse populations is warranted. As well, we used only one method to determine arterial stiffness, carotid ufPWV, and did not measure other carotid stiffness parameters, such as the Bramwell–Hill equation, to further illustrate arteriosclerosis.

## Conclusions

In conclusion, GDF15 proved to be a valuable predictor of arteriosclerosis in patients with OSA. It exhibited superior accuracy in identifying arteriosclerosis compared to clinical risk factors and other serum biomarkers, such as AOPP and IL-6. Moreover, a comprehensive evaluation of clinical risk factors, including age, gender, body mass index, SBP, FBG, and smoking, along with serum biomarkers like AOPP, IL-6, and GDF15, may have a more profound impact on assessing arteriosclerosis in patients with OSA. However, it is important to note that this study was a case–control study, and larger prospective studies are necessary to validate these findings.

### Supplementary Information

**Additional file 1.** Additional figures and tables.

## Data Availability

The datasets used during the current study are available from the corresponding author on reasonable request.

## Abbreviations

OSA: Obstructive sleep apnea
CVD: Cardiovascular diseases
AOPP: Advanced oxidative protein product
IL-6: Interleukin 6
GDF15: Growth differentiation factor 15
SpO_2_: Blood oxygen saturation
AHI: Apnea–hypopnea index
CCA: Common carotid artery
cIMT: Carotid intima–media thickness
PWV_BS: Pulse wave velocity_beginning of systole
PWV_ES: Pulse wave velocity_end of systole
ALT: Alanine transaminase
LDLC: Low-density lipoprotein cholesterol
HDLC: High-density lipoprotein cholesterol
FBG: Fasting blood glucose
OR: Odds ratios
CI: Confidence intervals
ROC: Receiver operating characteristic
AUC: Area under the curve
^c^NRI: Categorical net reclassification improvement
IDI: Integrated discrimination index
LOA: Limits of agreement
SBP: Systolic blood pressure
DBP: Diastolic blood pressure
ufPWV: Ultrafast pulse wave velocity
CPAP: Continuous positive airway pressure
PPV: Positive predictive value
NPV: Negative predictive value
LR (+): Positive likelihood ratio
LR (−): Negative likelihood ratio

## References

[1] Kapur VK, Auckley DH, Chowdhuri S, Kuhlmann DC, Mehra R, Ramar K (2017). Clinical practice guideline for diagnostic testing for adult obstructive sleep apnea: an American academy of sleep medicine clinical practice guideline. J Clin Sleep Med.

[2] Benjafield AV, Ayas NT, Eastwood PR, Heinzer R, Ip MSM, Morrell MJ (2019). Estimation of the global prevalence and burden of obstructive sleep apnoea: a literature-based analysis. Lancet Respir Med.

[3] Heilbrunn E, Ssentongo P, Chinchilli VM, Ssentongo AE (2020). Sudden death in individuals with obstructive sleep apnoea: protocol for a systematic review and meta-analysis. BMJ Open.

[4] Cao W, Luo J, Huang R, Xiao Y (2022). The association between sleep breathing impairment index and cardiovascular risk in male patients with obstructive sleep apnea. Nat Sci Sleep.

[5] Brown J, Yazdi F, Jodari-Karimi M, Owen JG, Reisin E (2022). Obstructive sleep apnea and hypertension: updates to a critical relationship. Curr Hypertens Rep.

[6] Seetho IW, Parker RJ, Craig S, Duffy N, Hardy KJ, Wilding JPH (2014). Obstructive sleep apnea is associated with increased arterial stiffness in severe obesity. J Sleep Res.

[7] Hvelplund Kristiansen M, Banghøj AM, Laugesen E, Tarnow L (2018). Arterial stiffness in people with Type 2 diabetes and obstructive sleep apnoea. Diabetic Med.

[8] Song F, Zou J, Song Z, Xu H, Qian Y, Zhu H (2020). Association of Adipocytokines With carotid intima media thickness and arterial stiffness in obstructive sleep apnea patients. Front Endocrinol.

[9] Theorell-Haglöw J, Hoyos CM, Phillips CL, Yee BJ, Melehan KL, Liu PY (2019). Associations between obstructive sleep apnea and measures of arterial stiffness. J Clin Sleep Med.

[10] Mitchell GF, Hwang SJ, Vasan RS, Larson MG, Pencina MJ, Hamburg NM (2010). Arterial stiffness and cardiovascular events: the Framingham Heart Study. Circulation.

[11] Seshadri S, Shokr H, Gherghel D (2022). Retinal Microvascular Abnormalities and Systemic Arterial Stiffness Are the First Manifestation of Cardiovascular Abnormalities in Patients with Untreated Moderate to Severe Obstructive Sleep Apnoea and with Low to Intermediate Cardiovascular Risk-A Pilot Study. Biomedicines..

[12] Orrù G, Storari M, Scano A, Piras V, Taibi R, Viscuso D (2020). Obstructive Sleep Apnea, oxidative stress, inflammation and endothelial dysfunction-An overview of predictive laboratory biomarkers. Eur Rev Med Pharmacol Sci.

[13] Witko-Sarsat V, Friedlander M, Capeillère-Blandin C, Nguyen-Khoa T, Nguyen AT, Zingraff J (1996). Advanced oxidation protein products as a novel marker of oxidative stress in uremia. Kidney Int.

[14] Perrone S, Laschi E, Buonocore G (2019). Biomarkers of oxidative stress in the fetus and in the newborn. Free Radical Biol Med.

[15] Ozben S, Huseyinoglu N, Hanikoglu F, Guvenc TS, Yildirim BZ, Cort A (2014). Advanced oxidation protein products and ischaemia-modified albumin in obstructive sleep apnea. Eur J Clin Invest.

[16] Yağmur AR, Çetin MA, Karakurt SE, Turhan T, Dere HH (2020). The levels of advanced oxidation protein products in patients with obstructive sleep apnea syndrome. Ir J Med Sci.

[17] Lacolley P, Regnault V, Laurent S (2020). Mechanisms of arterial stiffening: from mechanotransduction to epigenetics. Arterioscler Thromb Vasc Biol.

[18] Fiedorczuk P, Olszewska E, Polecka A, Walasek M, Mroczko B, Kulczyńska-Przybik A (2023). Investigating the Role of Serum and Plasma IL-6, IL-8, IL-10, TNF-alpha, CRP, and S100B Concentrations in Obstructive Sleep Apnea Diagnosis. Int J Mol Sci.

[19] Motamedi V, Kanefsky R, Matsangas P, Mithani S, Jeromin A, Brock MS (2018). Elevated tau and interleukin-6 concentrations in adults with obstructive sleep apnea. Sleep Med.

[20] Henry RM (2011). Interleukin 6 as therapeutic target in athero-/arteriosclerosis–small steps towards a new treatment paradigm in cardiovascular disease?. Atherosclerosis.

[21] Eddy AC, Trask AJ (2021). Growth differentiation factor-15 and its role in diabetes and cardiovascular disease. Cytokine Growth Factor Rev.

[22] Wollert KC, Kempf T, Wallentin L (2017). Growth differentiation factor 15 as a biomarker in cardiovascular disease. Clin Chem.

[23] Ho JE, Lyass A, Courchesne P, Chen G, Liu C, Yin X (2018). Protein biomarkers of cardiovascular disease and mortality in the community. J Am Heart Assoc.

[24] Sari K, Ede H, Kapusuz Gencer Z, Ozkiris M, Gocmen AY, Intepe YS (2015). The correlation of serum growth differentiation factor-15 level in patients with obstructive sleep apnea. Biomed Res Int.

[25] Tanaka T, Biancotto A, Moaddel R, Moore AZ, Gonzalez-Freire M, Aon MA (2018). Plasma proteomic signature of age in healthy humans. Aging Cell.

[26] Doerstling S, Hedberg P, Öhrvik J, Leppert J, Henriksen E (2018). Growth differentiation factor 15 in a community-based sample: age-dependent reference limits and prognostic impact. Upsala J Med Sci.

[27] Berry RB, Budhiraja R, Gottlieb DJ, Gozal D, Iber C, Kapur VK (2012). Rules for scoring respiratory events in sleep: update of the 2007 AASM Manual for the Scoring of Sleep and Associated Events Deliberations of the Sleep Apnea Definitions Task Force of the American Academy of Sleep Medicine. J Clin Sleep Med.

[28] Sprynger M, Rigo F, Moonen M, Aboyans V, Edvardsen T, de Alcantara ML (2018). Focus on echovascular imaging assessment of arterial disease: complement to the ESC guidelines (PARTIM 1) in collaboration with the Working Group on Aorta and Peripheral Vascular Diseases. Eur Heart J Cardiovasc Imaging.

[29] Yin LX, Ma CY, Wang S, Wang YH, Meng PP, Pan XF (2021). Reference values of carotid ultrafast pulse-wave velocity: a prospective, multicenter, population-based study. J Am Soc Echocardiogr.

[30] Lyle AN, Raaz U (2017). Killing Me unsoftly: causes and mechanisms of arterial stiffness. Arterioscler Thromb Vasc Biol.

[31] Vasan RS, Pan S, Xanthakis V, Beiser A, Larson MG, Seshadri S (2022). Arterial stiffness and long-term risk of health outcomes: the Framingham heart study. Hypertension.

[32] Williams B, Mancia G, Spiering W, Agabiti Rosei E, Azizi M, Burnier M (2018). 2018 ESC/ESH Guidelines for the management of arterial hypertension: The Task Force for the management of arterial hypertension of the European Society of Cardiology and the European Society of Hypertension: The Task Force for the management of arterial hypertension of the European Society of Cardiology and the European Society of Hypertension. J Hypertens.

[33] Messas E, Pernot M, Couade M (2013). Arterial wall elasticity: state of the art and future prospects. Diagn Interv Imaging.

[34] Zhu ZQ, Chen LS, Wang H, Liu FM, Luan Y, Wu LL (2019). Carotid stiffness and atherosclerotic risk: non-invasive quantification with ultrafast ultrasound pulse wave velocity. Eur Radiol.

[35] Marais L, Pernot M, Khettab H, Tanter M, Messas E, Zidi M (2019). Arterial Stiffness Assessment by Shear Wave Elastography and Ultrafast Pulse Wave Imaging: Comparison with Reference Techniques in Normotensives and Hypertensives. Ultrasound Med Biol.

[36] McEvoy RD, Antic NA, Heeley E, Luo Y, Ou Q, Zhang X (2016). CPAP for prevention of cardiovascular events in obstructive sleep apnea. N Engl J Med.

[37] Javaheri S, Barbe F, Campos-Rodriguez F, Dempsey JA, Khayat R, Javaheri S (2017). Sleep apnea: types, mechanisms, and clinical cardiovascular consequences. J Am Coll Cardiol.

[38] Chaddha A, Broytman O, Teodorescu M (2020). Effects of allergic airway inflammation and chronic intermittent hypoxia on systemic blood pressure. Am J Physiol Regul Integr Comp Physiol.

[39] Andersson C, Enserro D, Sullivan L, Wang TJ, Januzzi JL, Benjamin EJ (2016). Relations of circulating GDF-15, soluble ST2, and troponin-I concentrations with vascular function in the community: the Framingham Heart Study. Atherosclerosis.

[40] Wykretowicz A, Adamska K, Krauze T, Guzik P, Szczepanik A, Rutkowska A (2007). The plasma concentration of advanced oxidation protein products and arterial stiffness in apparently healthy adults. Free Radical Res.

[41] Kożuch M, Południewski M, Dąbrowski EJ, Tarasiuk E, Dobrzycki S (2022). Growth differentiation factor 15 as a predictor of the no-reflow phenomenon in patients with st-segment elevation myocardial infarction. J Clin Med.

[42] Maimaiti Y, Cheng H, Guo Z, Yu X, Tuohuti A, Li G (2023). Correlation between serum GDF-15 level and pulmonary vascular morphological changes and prognosis in patients with pulmonary arterial hypertension. Front Cardiovasc Med.

[43] Lind L, Wallentin L, Kempf T, Tapken H, Quint A, Lindahl B (2009). Growth-differentiation factor-15 is an independent marker of cardiovascular dysfunction and disease in the elderly: results from the Prospective Investigation of the Vasculature in Uppsala Seniors (PIVUS) Study. Eur Heart J.

[44] Rohatgi A, Patel P, Das SR, Ayers CR, Khera A, Martinez-Rumayor A (2012). Association of growth differentiation factor-15 with coronary atherosclerosis and mortality in a young, multiethnic population: observations from the Dallas Heart Study. Clin Chem.

[45] Bonaterra GA, Zügel S, Thogersen J, Walter SA, Haberkorn U, Strelau J (2012). Growth differentiation factor-15 deficiency inhibits atherosclerosis progression by regulating interleukin-6-dependent inflammatory response to vascular injury. J Am Heart Assoc.

