# Supplementary material for: Incremental values of AOPP, IL-6, and GDF15 for identifying arteriosclerosis in patients with obstructive sleep apnea
Source: Eur J Med Res. 2024 Feb 20;29:137. doi: 10.1186/s40001-024-01723-9 (PMC10877854; doi:10.1186/s40001-024-01723-9)
Supplement: Supplementary file 1 — Additional file 1. Additional figures and tables. [file 40001_2024_1723_MOESM1_ESM.docx]

**ADDITIONAL MATERIAL**

**Additional Figures and Figure Legend**


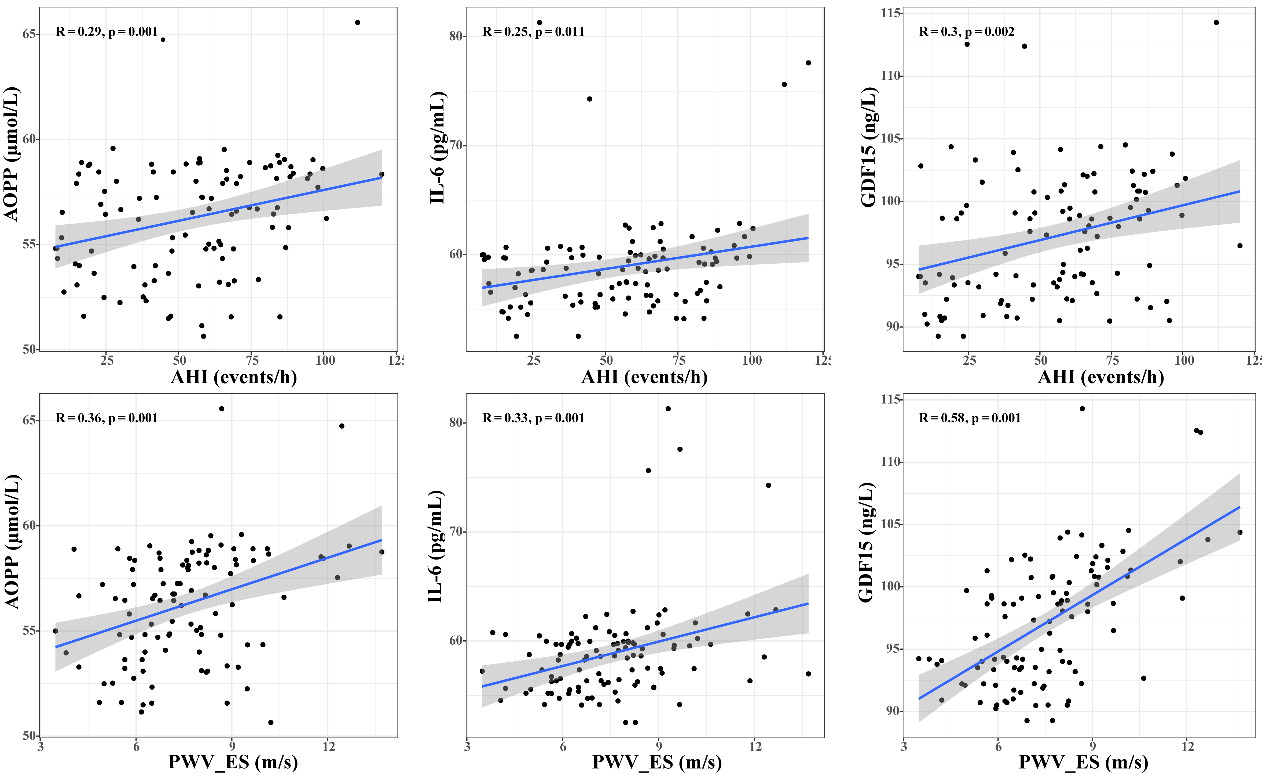


**Additional file 1: Figure S1. Correlation of serum biomarkers AOPP, IL-6 and GDF15 with AHI and PWV_ES in patients with OSA**

Abbreviations: AHI, Apnea-hypopnea index; AOPP, Advanced oxidation protein products; IL-6, Interleukin-6; GDF15, Growth differentiation factor 15; PWV_ES, pulse wave velocity at the end of systole.


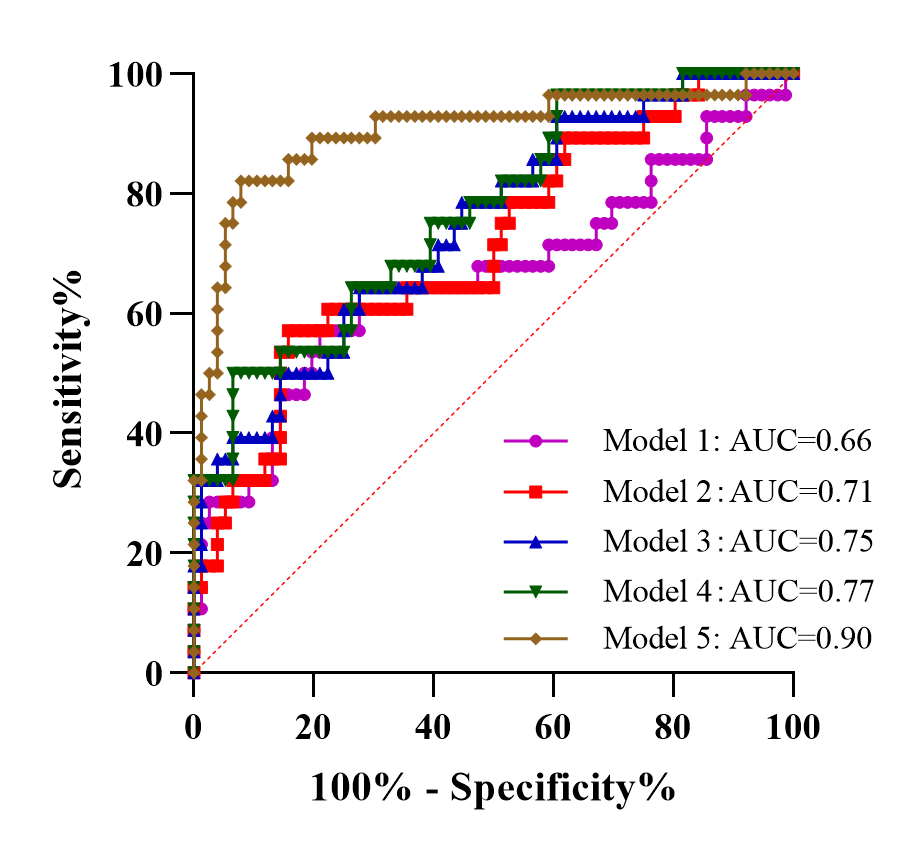


**Additional file 1: Figure S2. Receiver operating characteristic analysis of models to identify arteriosclerosis in patients with OSA.**

Model 1 included age, sex, Body mass index, SBP, FBG and smoking**;** Model 2 included Model 1 plus AHI; Model 3 included Model 2 plus AOPP; Model 4 included Model 3 plus IL-6; Model 5 included Model 4 plus GDF15.

Abbreviations: SBP, Systolic blood pressure; FBG, Fasting blood glucose; AHI, Apnea-hypopnea index; AOPP, Advanced oxidation protein products; IL-6, Interleukin-6; GDF15, Growth differentiation factor 15; AUC, Area under curve.

**Additional file 1: Table S1. A collinearity diagnostic on systolic blood pressure and diastolic blood pressure**

| **Collinearity Diagnostics^a^** | | | | | | |
| --- | --- | --- | --- | --- | --- | --- |
| Model | Dimension | Eigenvalue | Condition Index | Variance Proportions | | |
|  |  |  |  | (Constant) | SBP | DBP |
|  | 1 | .006 | 21.746 | .97 | .23 | .08 |
|  | 2 | .003 | 30.378 | .03 | .77 | .92 |
| a. Dependent Variable: arteriosclerosis | | | | | | |

**Additional file 1: Table S2. Backward stepwise logistic regression analysis was used to determine the sequence of markers adding to the model after clinical model.**

|  | | B | S.E. | Wald | *P* value | OR value | 95% CI | |
| --- | --- | --- | --- | --- | --- | --- | --- | --- |
|  |  |  |  |  |  |  | Lower | Upper |
| Step 1 | AHI | .003 | .013 | .075 | .784 | 1.003 | .979 | 1.029 |
|  | AOPP | .063 | .123 | .264 | .608 | 1.065 | .837 | 1.356 |
|  | IL-6 | .135 | .088 | 2.336 | .126 | 1.144 | .963 | 1.361 |
|  | GDF15 | .341 | .085 | 15.958 | .000 | 1.406 | 1.190 | 1.662 |
| Step 2 | AOPP | .073 | .118 | .379 | .538 | 1.076 | .853 | 1.357 |
|  | IL-6 | .144 | .083 | 2.995 | .084 | 1.155 | .981 | 1.359 |
|  | GDF15 | .343 | .085 | 16.362 | .000 | 1.409 | 1.194 | 1.664 |
| Step 3 | IL-6 | .144 | .081 | 3.175 | .075 | 1.155 | .986 | 1.354 |
|  | GDF15 | .354 | .084 | 17.754 | .000 | 1.424 | 1.208 | 1.679 |

Abbreviations: AHI, Apnea-hypopnea index; AOPP, Advanced oxidation protein products; IL-6, Interleukin-6; GDF15, Growth differentiation factor 15; OR, Odd ratio; CI, Confidence interval.

**Additional file 1: Table S3. Arteriosclerosis reclassification of AHI and serum biomarkers over the clinical models.**

|  | cNRI | 95% CI | *p* value | IDI | 95% CI | *p* value |
| --- | --- | --- | --- | --- | --- | --- |
| Model 1 | - | - | - | - | - | - |
| Model 2 | 0.19 | -0.03 to 0.41 | 0.09 | 0.01 | -0.00 to 0.09 | 0.06 |
| Model 3 | 0.05 | -0.13 to 0.22 | 0.58 | 0.05 | -0.01 to 0.10 | 0.08 |
| Model 4 | 0.04 | -0.11 to 0.18 | 0.63 | 0.06 | -0.01 to 0.12 | 0.06 |
| Model 5 | 0.31 | 0.11 to 0.51 | 0.01 | 0.16 | 0.10 to 0.24 | 0.01 |

Note: cNRI category risk: less than 30%, 30-60%, and greater than 60%.

Model 1 included age, gender, Body mass index, SBP, FBG and smoking.

Model 2 included Model 1 plus AHI.

Model 3 included Model 2 plus AOPP.

Model 4 included Model 3 plus IL-6

Model 5 included Model 4 plus GDF15

Abbreviations: SBP, systolic blood pressure; FBG, fasting blood glucose; AHI, Apnea-hypopnea index; AOPP, Advanced oxidation protein products; IL-6, Interleukin-6; GDF15, Growth differentiation factor 15; CI, Confidence interval; NRI, Net reclassification improvement; IDI, Integrated discrimination index.
